# The predictive value of computerized tomography‐assessed sarcopenia for complicated appendicitis in geriatric patients

**DOI:** 10.1002/agm2.12259

**Published:** 2023-06-30

**Authors:** Ali Cihat Yildirim, Şahinde Atlanoğlu, Mehmet Ali Gedik, Sezgin Zeren, Mehmet Fatih Ekici

**Affiliations:** ^1^ General Surgery Department Kutahya Health Sciences University Kutahya Turkey; ^2^ Radiology Department Kutahya Health Sciences University Kutahya Turkey

**Keywords:** appendicitis, geriatrics, perforated, sarcopenia

## Abstract

**Objective:**

Geriatric patients have more complicated appendicitis, which leads to higher morbidity and mortality rates. Sarcopenia has been shown to have a negative impact on patients undergoing surgery. This study aims to reveal the predictive value of computerized tomography‐assessed (CT‐assessed) sarcopenia for complicated appendicitis in geriatric patients.

**Methods:**

One‐hundred fifty‐four patients’ with acute appendicitis age, gender, co‐morbidities, appendicitis status, and body mass index (BMI) values were analyzed. The skeletal muscle index (SMI) and related measurements were evaluated.

**Results:**

Fifty‐two percent of the patients had complicated, and 48% had uncomplicated appendicitis. There was a statistically significant difference between uncomplicated and complicated cases regarding BMI, SMI, and muscle area values (*P* < 0.05). The cutoff point by Receiver Operating Characteristic Curve analysis was conducted for SMI and showed 71% sensitivity and 52% specificity (*P* = 0.042). Multivariate analysis has shown that comorbidities are significantly more associated with complicated appendicitis than sarcopenia.

**Conclusion:**

Geriatric patients with lower BMI, decreased muscle area, and CT‐detected sarcopenia have an increased risk of complicated appendicitis. Comorbidities are also important risk factors. Surgeons should be aware of factors leading to complicated appendicitis, which may cause higher morbidity and mortality rates in elderly patients.

## Graphical abstract

Geriatric patients have more complicated appendicitis, which leads to higher morbidity and mortality rates. According to our results, there was a statistically significant difference between uncomplicated and complicated cases in terms of body mass index (BMI), skeletal muscle index, and muscle area values (*P* < 0.05). Geriatric patients who have lower BMI, decreased muscle area, and computerized tomography‐detected sarcopenia have an increased risk of complicated appendicitis.

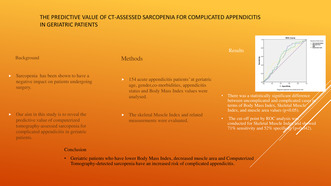

## 1 INTRODUCTION

Acute appendicitis is one of the most common surgical pathologies. The average lifetime risk of having acute appendicitis is 7% to 8%. Acute appendicitis occurs in 15% of patients above the age of 50 years who have acute abdominal pain and 5% of those above the age of 65 years; accordingly, it is not an uncommon pathology in the elderly population.^1^, ^2^, ^3^

Traditionally, “elderly” or “geriatric” was defined as a chronological age of 65 years or older. However, the evidence for this chronological point of view is still unclear.^4^ Geriatric patients with acute appendicitis are at more risk than younger patients. First, possible comorbidities and frailty can result in diagnostic delays. Second, geriatric patients tend to have more complicated appendicitis, as the percentage presenting with perforation or abscesses ranges from 18% to 70%. Hence, they have higher morbidity and mortality rates than younger patient groups (8% for the elderly vs 0%–1% for younger patients).^2^, ^3^

Sarcopenia is the decline in lean skeletal muscle mass and function, primarily encountered in advanced age. It has an overall incidence of 10% in men and women worldwide. Moreover, its incidence rises by 15% per decade over the age of 70 years.^5^ The decreased strength and function of the muscle in sarcopenia is progressive and generalized, leading to impaired physical capacity and negatively affecting the quality of life and postoperative mortality. There are several methods to measure sarcopenia, including radiological investigations, such as computerized tomography (CT) and magnetic resonance imaging (MRI), which are among the techniques used to evaluate muscle quantity and quality. The psoas muscle is an indicator of the skeletal muscle mass index (SMI) analyzed on CT‐assessed sarcopenia.^6^

Sarcopenia has shown a negative impact on both elective cancer surgeries and emergency surgeries regarding morbidity and mortality.^7^ Consequently, sarcopenia has been used as a prognostic tool to identify those patients who may suffer adverse events after elective or emergency surgery.

In this study, our primary aim was to reveal the predictive value of CT‐assessed sarcopenia for complicated appendicitis in geriatric patients. We also analyzed the predictive value of body mass index (BMI) and the other CT‐assessed parameters of muscle area, mesenteric fatty tissue area, and single slice total area to diagnose complicated appendicitis.

## 2 MATERIALS AND METHODS

### 2.1 Patient data collection

After approval was gained from the local ethical committee (Kutahya Health Sciences University Local Ethical Committee Approval Number: 2021/07‐06), 154 patients with acute appendicitis of geriatric age (65 years or older) who were operated on in our general surgery clinic between January 2018 and October 2020 and had a contrast‐enhanced abdominal CT scan prior to surgery were included in this retrospective research. Of 159 patients, five without CT scans were excluded from the study.

Patient demographic and anthropometric data (age, gender, weight, and height) were obtained from patient files. Comorbidities of cardiovascular disease, hypertension, diabetes mellitus, and smoking history of the patients were also included in the data. BMI was calculated from the patient's weight in kilograms and height in meters squared (kg/m^2^). Because the study was retrospective, written informed consent was waived.

### 2.2 CT scan

All CT scans were performed before the surgical operation and were part of the diagnostic routine. The scans were conducted with a 16‐slice multidetector CT scanner (Aquilion, Toshiba Medical Systems). Images were contrast‐enhanced and acquired at the portal venous phase with a 65‐second delay after intravenous contrast material administration in the supine position. When subjects hold their breath, images can be obtained from the heart level to the symphysis pubis. Scanning parameters are 120 kVp, the mAs value with automatic tube current modulation, FOV of 370, and section thickness 5 mm. Voxel sizes are 0.75 × 0.75 × 5 mm.

### 2.3 CT measurements

To evaluate complicated and non‐complicated appendicitis, two radiologists separately assessed the existence of 10 CT signs of complicated appendicitis (abscess, extraluminal air, luminal air, extraluminal appendicolith, luminal appendicolith, moderate‐to‐severe peri‐appendiceal fat stranding, peri‐appendiceal fluid, ileus, ascites, and contrast enhancement defects of the appendiceal wall).^8^ Furthermore, the intra‐operative evaluation of the appendicitis status has been retrospectively examined to coordinate the radiological signs of complicated appendicitis, so complicated cases detected on CT scans were also confirmed by operative notes.

Two radiologists executed measurements (reader one, author M.A.G., with 8 years of experience in radiology; reader two, author Ş.A., with 10 years of experience in radiology) from within the 3D slicer program. The first reader conducted all 154 patient measurements. Twenty‐seven randomly selected patients were re‐evaluated by the first reader and evaluated twice by the second reader at an interval of 1 month to estimate the intra‐observer and interobserver reliability.

**FIGURE 1:**
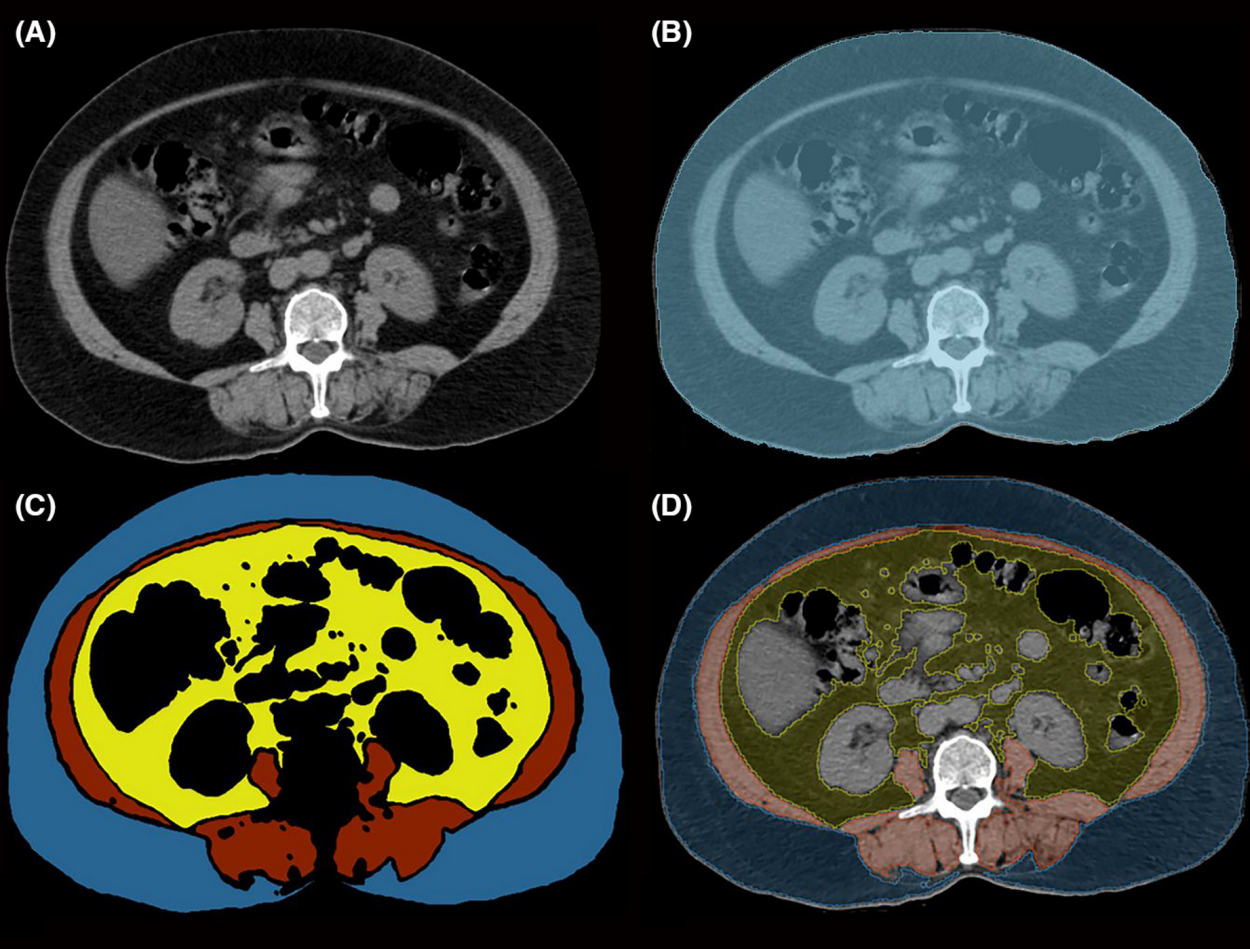
Axial unenhanced CT scan at L3 vertebrae mid‐corpus level. (A) raw image; (B) all tissues segmented (single slice); (C) Mask images of segmentation, blue represents subcutaneous fatty tissue, red muscle tissue and yellow mesenteric fatty tissue; (D) Segments on raw CT image. CT, computed tomography.

The voxel size of all patients (0.75 × 0.75 × 5.00 mm) was resampled to 1 × 1 × 1 mm with the Resample Scalar Volume module. The third lumbar vertebra is detected in the sagittal plane, and volume data are cropped with the crop volume module to include only the third lumbar vertebrae. In the sagittal plane, a single slice located in the middle of the third vertebrae is selected, and all measurements of each patient by all readers are conducted on this axial plane CT slice. The slice is divided into four segments: total cross‐sectional area (single slice total area), subcutaneous fatty tissue, muscle tissue, and visceral fatty tissue areas (mesenteric fatty tissue area). The total cross‐sectional area is used as a mask, and the other three segments are painted in that mask. The total cross‐sectional area is segmented with a paint tool. Different segments are painted mainly with semi‐automatic flood‐filling tools. Paint, draw, erase, logical operators, islands, and smoothing tools are used for fine adjustment. The area (cm^2^), maximum, minimum, mean, median, and standard deviation values of the Hounsfield unit for each segment are computed with the segment statistics module (Figure 1).

The SMI was used as the preferred CT‐assessed sarcopenia index. It was obtained from the skeletal muscle area and adjusted for stature (cm^2^/m^2^) on CT images at the L3 vertebra level.

### 2.4 Statistical analysis

The IBM Statistical Package for the Social Sciences for Windows, version 20.0 (SPSS Inc..) was used for statistical analysis and evaluation of the findings obtained in the study.

Descriptive data on age, gender, comorbidities, BMI, and appendicitis status (complicated or uncomplicated appendicitis, as evaluated by CT) were calculated. Furthermore, CT‐assessed sarcopenia parameters were calculated, including SMI, muscle area, mesenteric fatty tissue area, and single slice total area.

A Kolmogorov–Smirnov test determined the normality of the continuous variables. Where applicable, data were shown as mean ± standard deviation or median (minimum–maximum). The differences between groups were compared using the independent samples *t* test or the Mann–Whitney *U* test, where appropriate. Categorical data were analyzed by Pearson's chi‐square test, where appropriate. The cutoff values of the groups' discrimination parameters were determined using the receiver operating characteristic (ROC) curve analysis. The sensitivity and specificity for each outcome under study were plotted at each value, thus generating an ROC curve. A *P* value of < 0.05 and <0.10 was considered statistically significant where applicable. A multivariate logistic regression model for complicated appendicitis was generated using a backward stepwise method to assess whether sarcopenia predicted complicated appendicitis independently of comorbidities.

## 3 RESULTS

Of the 154 geriatric patients, 62% were men (n = 95) and 38% (n = 59) were women; 52% of the patients included in the study were in the complicated (*n* = 80) and 48% (n = 74) in the uncomplicated category. There was no difference in gender distribution between uncomplicated and complicated cases (*P* = 0.654). The ratio of male individuals was higher in both categories.

Table 1 shows the statistical relationship between the variables and appendicitis status.

**TABLE 1:** Statistical relationship between the variables and appendicitis status.

	Non‐complicated (n = 74)				Complicated (n = 80)				*P* value
	Min	Max	Median	IQR	Min	Max	Median	IQR	
Age, y	65	93	72	11.25	65	90	73	10.75	0.712
SMI	19.13	80.97	45.14	12.03	27.18	65.16	41.99	16.02	0.042*
Subcutaneous fatty tissue area	13.65	387.46	217.24	140.40	29.76	706.25	178.81	131.96	0.068**

	Min	Max	Mean	SD	Min	Max	Mean	SD	*P* value
BMI	19.25	37.98	27.29	4.50	17.52	35.45	25.28	3.64	0.003*
Muscle area	60.61	217.78	125.52	25.07	74.91	180.76	116.87	25.44	0.035*
Mesenteric fatty tissue area	9.98	389.30	176.06	88.41	21.05	489.59	170.25	93.64	0.693
Single slice total area	281.21	1432.12	724.15	248.17	236.19	1420.85	692.01	218.54	0.394

Gender	Female n (%)		Male n (%)		Female n (%)		Male n (%)		*P* value
	27 (36.5)		47 (63.5)		32 (40)		48 (60)		0.654

*Note*: Age, SMI, subcutaneous fatty tissue area: Mann–Whitney *U* test; BMI, muscle area, mesenteric fatty tissue area, single slice total area: independent samples *t* test; Gender: chi‐square tests.

Abbreviations: BMI, body mass index; IQR, interquartile range; Max, maximum; Min, minimum; SD, standard deviation; SMI, skeletal muscle index.

*P < 0.05; **P < 0.10.

There is a statistically significant difference between uncomplicated and complicated cases regarding BMI, SMI, and muscle area values with a 0.05 margin of error. In terms of subcutaneous fatty tissue area value, there is a statistically significant difference with a 0.10 margin of error between uncomplicated and complicated cases. No statistically significant difference exists between uncomplicated and complicated cases relative to age, mesenteric fatty tissue area, and single‐slice total area values.

The most appropriate cutoff point was applied by ROC analysis to BMI, sarcopenia index, and muscle area variables, which had a statistically significant difference with a 0.05 margin of error between uncomplicated and complicated cases.

Receiver operating characteristic curve analysis was performed with data from the sarcopenia index results (Table 2). The cutoff point for the sarcopenia index was determined as 41.62 cm^2^/m^2^, with a sensitivity of 71% and a specificity of 52%. The obtained area under the curve (AUC) value was 0.60 (*P* = 0.042).

**TABLE 2:** Results of ROC curve analysis of the variables.

	Cutoff value	Sensitivity	Specificity	AUC *P* value
SMI (cm^2^/m^2^)	41.62	0.71	0.52	0.60–0.042*
Muscle area (cm^2^)	115.66	0.72	0.54	0.60–0.032*
BMI (kg/m^2^)	24.97	0.69	0.52	0.62–0.010*

Abbreviations: AUC, area under curve; BMI, body mass index; ROC, receiver operating characteristic curve; SMI, skeletal muscle index.

* *P* < 0.05.

When ROC analysis was performed with the data of the muscle area results, the cutoff point of the muscle area was determined as 115.66 cm^2^ with 72% sensitivity and 54% specificity. The AUC value was 0.60 (*P* = 0.032).

**FIGURE 2:**
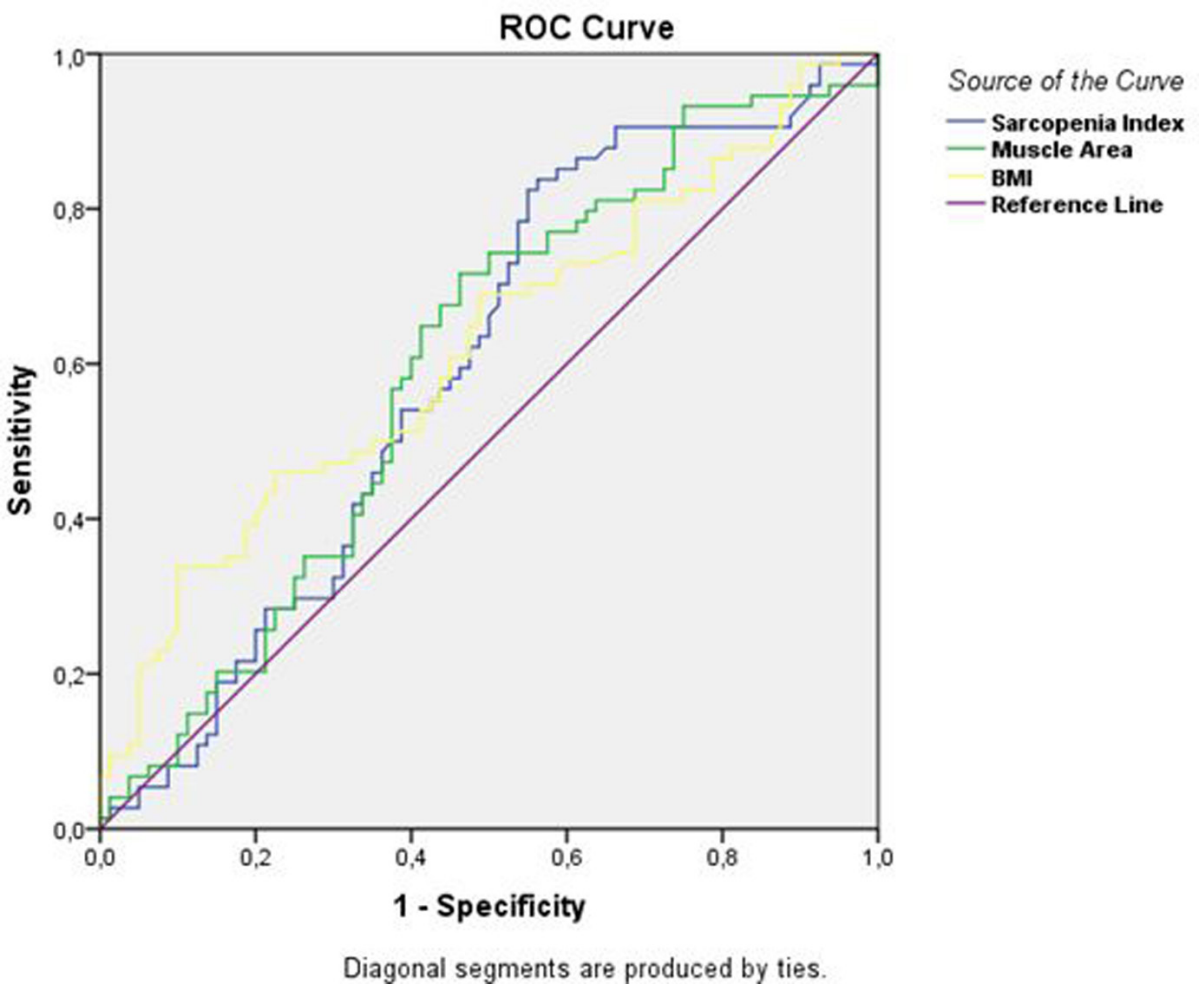
Receiver operating characteristic curve analysis of the sarcopenia index (SMI cm^2^/m^2^), muscle area, and BMI for predicting complicated appendicitis. BMI, body mass index; ROC, receiver operating characteristic curve; SMI, skeletal muscle index.

When an ROC analysis was performed with the data of BMI results, the cutoff point for BMI was determined as 24.97 kg/m^2^ with 69% sensitivity and 52% specificity. The AUC value was also calculated as 0.62 (*P* = 0.010; Figure 2).

Furthermore, the combined effect of sarcopenia and the comorbidities of cardiovascular disease, diabetes mellitus (DM), hypertension, and smoking history on the dependent variable appendicitis status were analyzed by logistic regression analysis backward stepwise method. Accordingly, cardiovascular disease, DM, and smoking history variables were found to be statistically significant (*P* < 0.05).

According to the detected cutoff threshold of sarcopenia (41.62 cm^2^/m^2^), the risk of complicated appendicitis was 1.873 times higher in patients with sarcopenia than in those without sarcopenia. However, it was not found to be statistically significant in multivariate analysis (*P* > 0.05; Table 3).

**TABLE 3:** Examination of independent variables affecting appendicitis status by logistic regression analysis.

Multivariate	OR	95% CI for OR	*P* value
	Lower	Upper	
Step 1			
Sarcopenia index (cm^2^/m^2^)	1.873	(0.794–4.421)	0.152
Cardiovascular disease	11.472	(4.728–27.833)	0.000*
DM	2.982	(1.245–7.140)	0.014*
Smoking	3.070	(1.262–7.472)	0.013*
HT	2.069	(0.874–4.898)	0.098
Step 2			
Cardiovascular disease	12.667	(5.267–30.463)	0.000*
DM	2.842	(1.201–6.727)	0.017*
Smoking	2.923	(1.213–7.041)	0.017*

Abbreviations: CI, confidence interval; DM, diabetes mellitus; HT, hypertension; OR, odds ratio.

* *P* < 0.05, Binary logistic regression analysis (backward stepwise method).

## 4 DISCUSSION

Our study presents the first analysis of the predictive value of CT‐assessed sarcopenia for complicated appendicitis in the literature. The study shows that CT‐assessed sarcopenia index SMI, muscle volume, and BMI can predict complicated appendicitis in geriatric patients.

Geriatric age is among the factors associated with complicated appendicitis.^9^ Complicated appendicitis is associated with comorbidities, such as anemia, cardiac disease, and chronic renal disease in geriatric patients.^10^

Higher rates of complicated appendicitis in geriatric patients are also attributed to risk factors, such as vascular sclerosis, fibrotic narrowing of the lumen, and fat infiltration on the muscular layer of the appendix. As a result, complications, such as perforation, tend to occur more easily.^11^ Moreover, there might also be a diagnostic delay related to an inability to sense pain and laboratory abnormalities of comorbidities in advanced age.^12^ Fifty‐two percent of the patients included in this study were in the complicated appendicitis category. The patient population in our study was of geriatric age, so the complicated appendicitis ratio was higher than that seen in the general population.

The term “sarcopenia” has been previously used to describe the loss of muscle with increased age; however, according to new definitions, physical performance and muscle strength are equally important as a decrease in muscle volume. Its multifactorial pathogenesis includes chronic metabolic changes, low BMI, and reduced activity and protein intake. The factors usually combine, resulting in dysfunction of muscle mass function and strength.

Consequently, it may be better interpreted as a syndrome that may be presented in different degrees in different patient cohorts.^13^ As the degree of muscle volume decreases, adverse events seem more highly related to decreased muscle strength or function.^14^

Several validated methods exist to measure sarcopenia, such as dual X‐ray absorptiometry, bioelectric impedance analysis, MRI, and CT scanning. Of these techniques, CT is considered the gold standard to diagnose sarcopenia with a range of minimal errors (1%–4%).^15^ Although it is more expensive than anthropometric methods and poses a greater radiation exposure than the other techniques, it provides high accuracy and reproducible results. It also enables the evaluation of lean body mass and visceral and subcutaneous fat tissues concurrently.^16^

In our retrospective study, we chose SMI to assess sarcopenia on CT. In this technique, the psoas muscle at the L3 vertebra level on cross‐section imaging was analyzed as representative of the total body muscle mass, which gave information about muscle quantity and quality. The distinction among different tissues, such as muscle and fat, was assessed by Hounsfield units.^14^

In general, CT has been widely used in geriatric patients presenting with emergency abdominal pain, especially in differential diagnosis. CT results in the elderly could be highly influential on the direction of the therapeutic process.^17^ Thus, analyzing sarcopenia simultaneously with an index CT might have several benefits, such as predicting complicated appendicitis. In our study, we used the pre‐operative contrast‐enhanced abdominal CT to differentiate appendicitis status and analyze the sarcopenia at the same time.

Recently, sarcopenia has been analyzed based on the surgical outcomes of patients who have undergone elective cancer surgery or emergency abdominal surgery. In general, CT‐assessed sarcopenia has been shown to be a risk factor in gastrointestinal oncology patients' short‐ and long‐term outcomes. In geriatric patients with colorectal cancer, sarcopenia has been not only associated with postoperative complications and poor long‐term outcomes but can also predict pre‐operative nutritional risks. Therefore, it can guide treatment and follow‐up strategies. Studies have shown that sarcopenia among patients undergoing exploratory laparotomy has been associated with higher morbidity, mortality, hospital length of stay, and undesirable discharge rates.^18^ It is also associated with higher mortality rates in geriatric patients who underwent emergency abdominal surgery during the 1‐year postoperative follow‐up period.^19^

Our study's results align with other research and show that sarcopenia is important in predicting complicated appendicitis to some degree. However, according to multivariate regression analysis, comorbid conditions like cardiovascular disease, diabetes, and a history of smoking were more critical than sarcopenia in predicting complicated appendicitis.

The association between sarcopenia and complicated appendicitis examined in the study cannot be interpreted as a direct cause‐and‐effect relationship. The presence of general risk factors already present in patients with sarcopenia more often co‐exists with conditions that also cause complicated appendicitis.

Because it was a retrospective study, the inability to identify all comorbid conditions and their severity in our study and the method of assessment of sarcopenia may have been practical.

In our patient cohort, the mean BMI of geriatric patients with complicated appendicitis was 25.28 kg/m^2^, whereas with uncomplicated cases, it was 27.29 kg/m^2^. Although statistical significance exists between the mean BMI levels of the two groups, it might not be interpreted as clinical significance. According to the National Institute of Health and World Health Organization classifications, a BMI between 25 and 29.9 kg/m^2^ is considered overweight. BMI is a statistical index for estimating body fat in both genders and is commonly used. However, due to individual and geographic variations, it is still insufficient to classify a person as obese or malnourished.^20^ Further, it does not differentiate between body lean mass and body fat mass. It is an easily obtainable metric but does not correlate solely with a patient's morbidity and mortality when considering many variables, including genetic and environmental confounders.^21^ In the literature, BMI as an indicator of nutritional status is associated with different surgical outcomes in different patient cohorts. Obese geriatric patients who have undergone emergency surgery have higher morbidity and lower mortality rates than their normal‐BMI counterparts.^22^ Another study showed that higher BMI was an independent risk factor for early anastomotic leakage after colorectal surgery.^23^ In our study, the mean BMI values of the two groups can both be categorized as overweight. Although geriatric obesity is a growing concern worldwide, Turkey has one of the highest obesity risks in Europe, and sarcopenic obesity was seen in 11% of one geriatric patient cohort; however, in our geriatric patients, the mean BMI of the two groups did not support any association between sarcopenia and obesity.^24^

Additionally, our study results show that the mean muscle area assessed by CT between groups was statistically significant. The mean value for the uncomplicated appendicitis group was 125.52 cm^2^, whereas for the complicated appendicitis group, it was 116.87 cm^2^. This significance might be co‐related to the significant difference between groups of the sarcopenia index.

There is a statistically significant difference with a 0.10 margin of error between uncomplicated and complicated cases regarding subcutaneous fatty tissue area values. This significance is correlated with the significant difference in the mean BMI values of geriatric patients with different appendicitis statuses.

In our study, our patient cohort did not differ significantly regarding gender for the prediction of complicated appendicitis, so we did not use previous cutoff values for the CT‐assessed sarcopenia index. The cutoff value of sarcopenia, assessed by ROC analysis, was 41.62 cm^2^/m^2^, showing 71% sensitivity and 52% specificity.

Moreover, the cutoff value of the muscle area assessed by ROC analysis was 115.66 cm^2^, which has shown 72% sensitivity and 54% specificity.

Finally, when an ROC analysis was performed with the data yielded by BMI results, the cutoff point for BMI was determined as 24.97 kg/m^2^, with 69% sensitivity and 52% selectivity. The AUC value was also calculated as 0.62 (*P* = 0.010). The three cutoff values have similar sensitivity and specificity.

There are several scoring systems for diagnosing acute appendicitis. One of the most used methods, the Alvarado score, has high sensitivity and specificity values, especially to exclude acute appendicitis when the score is < 5. However, few studies have analyzed the scoring systems in geriatric patients. Hence, no scoring system exists to discriminate complicated from uncomplicated appendicitis in such patients. Recently, a CT‐based acute appendicitis severity index has been found to be a reliable parameter predicting complicated appendicitis.^25^ Furthermore, using imaging analysis together with the clinical scoring systems may enable better differentiation in cases of complicated and uncomplicated appendicitis.^26^

According to our study results, CT‐assessed sarcopenia might predict complicated appendicitis in geriatric patients with a moderate degree of sensitivity and specificity and be used separately or in combination with diagnostic scoring systems. In multivariate analysis, comorbid conditions were more significantly associated with complicated appendicitis than sarcopenia. This study showed the need for prospective studies involving a larger number of patients to demonstrate the actual predictive value of sarcopenia.

## 5 CONCLUSION

Geriatric patients with lower BMI, decreased muscle area, and CT‐detected sarcopenia have an increased risk of complicated appendicitis. Surgeons should be aware of the factors that can lead to complicated appendicitis, which may result in higher morbidity and mortality rates among the elderly.

## AUTHOR CONTRIBUTIONS

*Concept:* Yildirim, Atlanoğlu, Gedik, Zeren, and Ekici. *Design:* Yildirim, Atlanoğlu, and Gedik. *Supervision:* Yildirim, Zeren, and Ekici. *Resource:* Yildirim, and Zeren. *Materials:* Yildirim, Ekici, and Zeren. *Data:* Yildirim, Ekici, and Zeren. *Analysis:* Atlanoğlu and Gedik. *Literature search:* Yildirim and Gedik. *Writing:* Yildirim, Gedik, and Atlanoğlu. *Critical revision:* Zeren and Ekici.

## FUNDING INFORMATION

The authors declared that this study has received no financial support.

## CONFLICT OF INTEREST STATEMENT

The authors declare no conflicts of interest.

## ETHICS STATEMENT

The study was approved by the local ethics committee (Kutahya Health Sciences University Local Ethical Committee Approval Number: 2021/07‐06) and certify that the study was performed in accordance with the ethical standards as laid down in the 1964 Declaration of Helsinki and its later amendments or comparable ethical standards.
